# On the Mechanics of Cardiac Function of *Drosophila* Embryo

**DOI:** 10.1371/journal.pone.0004045

**Published:** 2008-12-24

**Authors:** Mingming Wu, Thomas N. Sato

**Affiliations:** 1 The Sibley School of Mechanical and Aerospace Engineering, and the Department of Chemical and Biomolecular Engineering, Cornell University, Ithaca, New York, United States of America; 2 Department of Cell and Developmental Biology, Weill Medical College of Cornell University, New York, New York, United States of America; Tufts University, United States of America

## Abstract

The heart is a vital organ that provides essential circulation throughout the body. Malfunction of cardiac pumping, thus, leads to serious and most of the times, to fatal diseases. Mechanics of cardiac pumping is a complex process, and many experimental and theoretical approaches have been undertaken to understand this process. We have taken advantage of the simplicity of the embryonic heart of an invertebrate, *Drosophila melanogaster*, to understand the fundamental mechanics of the beating heart. We applied a live imaging technique to the beating embryonic heart combined with analytical imaging tools to study the dynamic mechanics of the pumping. Furthermore, we have identified one mutant line that exhibits aberrant pumping mechanics. The *Drosophila* embryonic heart consists of only 104 cardiac cells forming a simple straight tube that can be easily accessed for real-time imaging. Therefore, combined with the wealth of available genetic tools, the embryonic *Drosophila* heart may serve as a powerful model system for studies of human heart diseases, such as arrhythmia and congenital heart diseases. We, furthermore, believe our mechanistic data provides important information that is useful for our further understanding of the design of biological structure and function and for engineering the pumps for medical uses.

## Introduction

The heart is the center of the circulatory system, which is one of the vital organs for survival of organisms. Extensive experimental and theoretical approaches have been undertaken to understand the mechanics of human heart function [1]. Such studies have contributed to developing many models that explain some functional aspects of normal and pathological heart [2]. The major hindrance to studying human heart is its complexity of structure and function. The analyses of hearts of other organisms that possess structurally simpler hearts may provide useful insights into understanding some of the fundamental aspects of the mechanics of human heart.

One such model organism is zebrafish, which is one of the most popular model organisms for understanding the genetic and molecular basis of developmental and physiological processes. Zebrafish is a vertebrate and its heart consists of two chambers, ventricle and atrium [3], [4]. Genetic analyses of zebrafish heart revealed many fundamentally important molecular pathways underlying its formation and function, which are conserved among all vertebrates including human [3]–[5]. In contrast to the wealth of such molecular information regarding the formation and function of the zebrafish heart, very little is known about the mechanics of cardiac function. Recently, by taking the advantage of its transparency and accessibility to experimental manipulations and live imaging, zebrafish embryos have been used to study the mechanics of cardiac pumping [6], [7]. By applying a high-speed live imaging technology to the embryonic heart of zebrafish, it was demonstrated that the embryonic zebrafish heart is a dynamic suction pump, rather than a peristaltic pump as previously proposed.

**Figure 1:**
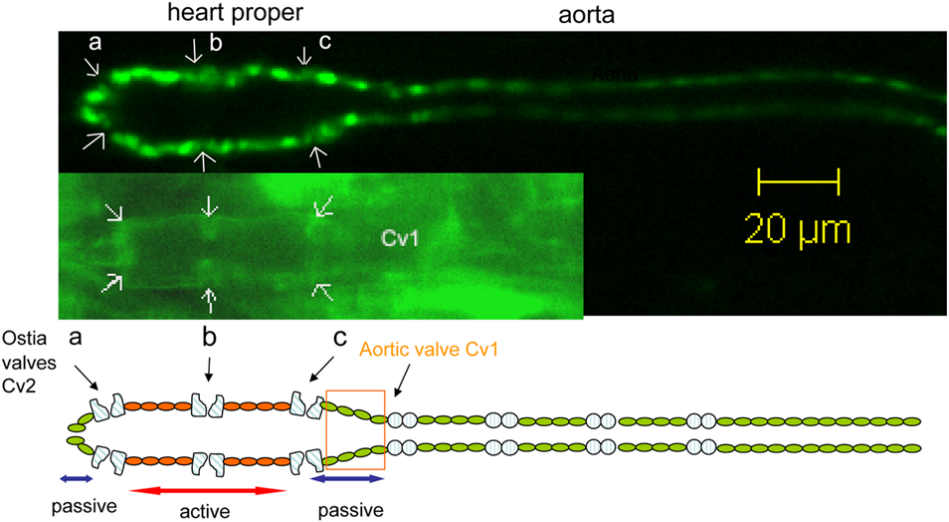
Structure of the tubular embryonic *Drosophila* heart and the aorta. The upper panel is a fluorescence image of a fruit fly embryo at stage 17, expressing a nuclear green fluorescent protein (GFP) marker in all the cardio blasts (toll-nGFP). The insert is the DMef2::Gal4;Twist::Gal4;UAS::actinGFP embryo showing morphologically distinguishable ostia cells (arrows). The lower panel illustrates the structure of the tube heart and the aorta consisting of two rows of cardiac cells (52 cells each row). Three pairs of ostia cells are spaced equally along the heart proper that serve as the inflow check valves. The orange box indicates where the outflow check valve is. We name it an aortic valve. This is derived from the heart beat function observed in our experiments. Whether the molecular details of the cells in the aortic valve region are different from the other cardiac cells are not known.

Although zebrafish heart provides a useful model system to study the mechanics of the most primitive chambered heart, it may be as well useful to study the mechanics of cardiac pumping of the heart of even simpler structure. *Drosophila* is one of the most popular invertebrate model organisms that have been used for centuries. The heart of *Drosophila* is a simple straight tube consisting of two rows of cardiac cells forming a linear tubular structure [8], [9]. During embryogenesis, a total of 52 cardiac precursor cells exist on each side of embryonic body separated by the dorsal midline axis, which come together to form a tubular structure referred to as dorsal vessel. Towards the end of embryogenesis, the posterior portion (heart proper) becomes wider than the anterior portion (aorta) (Figure 1). Soon after these two distinguishable structures appear, three sets of cardiac cells, each set consisting of four cells with two cells on each row, in the heart proper appears morphologically distinguishable from the rest of the cardiac cells (Figure 1). These cardiac cells of unique shape are called ostia and suspected to form channels for body fluid (hemolymph) to enter into the heart proper [10]. In addition to the differentiation of cardiac cells to the morphologically distinguishable cells, they also differentiate into molecularly distinguishable cells [8]–[10]. In the past couple two decades, genetic studies of developing *Drosophila* heart have uncovered evolutionarily conserved molecular pathways that specify the identity of cardiac cells [11]–[13]. More recently, such classical genetic studies have been successfully applied to gain insight into the molecular pathways that control the formation of tubular structure of the *Drosophila* heart [14]–[16]. Furthermore, the genetic approaches in studying the *Drosophila* heart also provided some important insights into the molecular mechanisms underlying the cardiac functions [17].

In contrast to the existence of such extensive knowledge on the molecular and genetic controls of cardiac formation and function, almost nothing is known about the pumping mechanics of the *Drosophila* heart. It is presumed that the major function of the *Drosophila* heart is to pump and distribute hemolymph throughout the body. It has been proposed that a valve structure that separates the aorta from the heart proper opens and closes, in coordination with openings/closings of ostia, to control unidirectional flow of hemolymph through the dorsal vessel [8], [10]. During the rhythmic contractions of the heart, the opening of the ostia allows the entry of hemolymph into and fills the heart proper as the aortic valve closes. As the ostia closes, the aortic valve opens allowing the hemolymph being pumped out through the aorta.

Although such description of pumping mechanics intuitively makes sense, the precise mechanical description of such pumping function is missing in the published literature. Therefore, we used a combination of live imaging of the beating *Drosophila* embryonic heart and analytical tools to gain insight into the mechanics of the beating *Drosophila* heart. In this report, we demonstrate several features of pumping mechanics of the beating embryonic heart of *Drosophila*. Our studies have also revealed a pumping mechanics of a simple tubular heart of invertebrate that surprisingly mimics an aspect of the beating human heart. Further, towards our understanding of the molecular basis of this pumping mechanics, we have also identified a genetic mutation that leads to an aberrant pumping mechanics.

## Results

### Sequential opening/closing of valves in the tubular heart of *Drosophila* embryo

To study the dynamics of the pumping mechanics of *Drosophila* heart in live, we used a transgenic *Drosophila* line, Toll-nGFP, where GFP is expressed in the nucleus of all individual cardiac cells [18]. The dynamics of the motion of each cardiac cell was imaged by a line-scanning high-speed confocal microscopy system, which was previously used for imaging the live embryonic heart of zebrafish [6].

**Figure 2:**
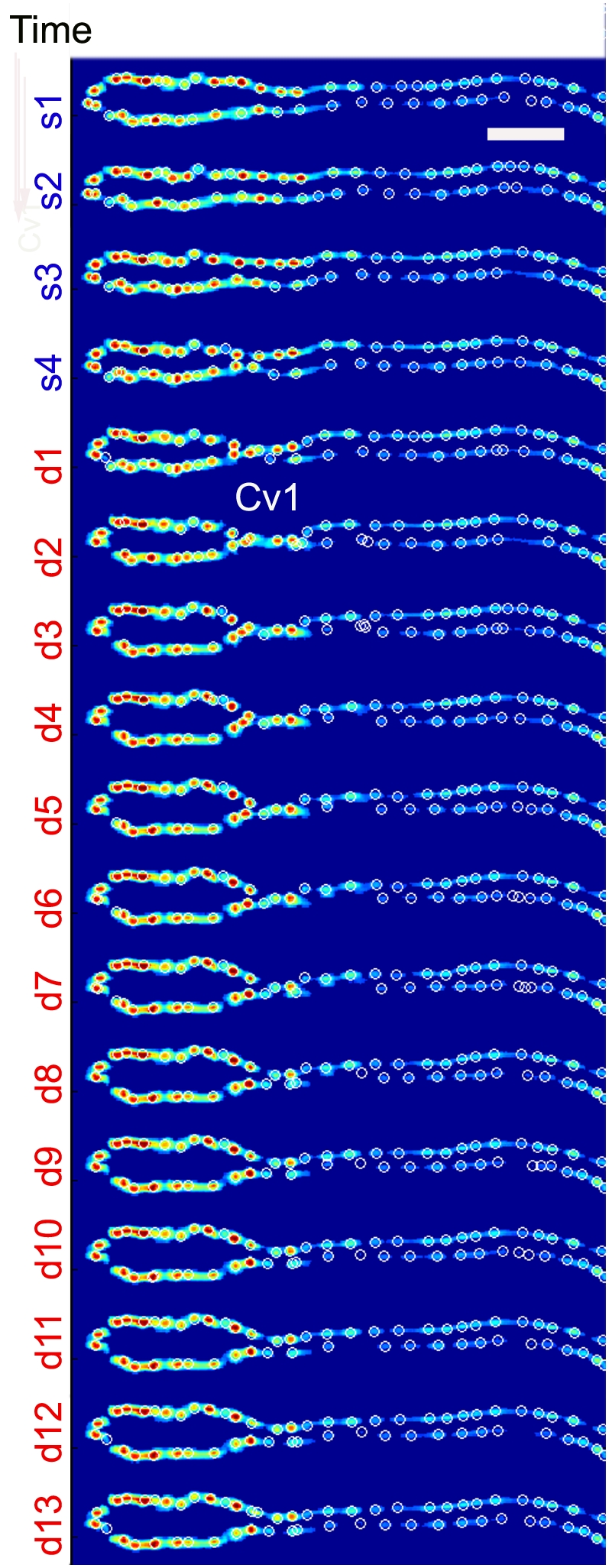
Tubular heart dynamics during one heart beat. This is a sequence of images of the tube heart (toll-nGFP) taken at 50 ms apart. The color spectrum from red to blue corresponds to emitted GFP light intensity from high to low. As shown, heart volume decreases during systolic phase (s1–s4), and increases during diastolic phase (s4-d13-s1). At the same time, the aortic valve Cv1 is open during systolic phase; while it is closed during diastolic phase. The ratio of the duration of the diastolic period to that of systolic is 5∶1. The period of the heart beat is 0.85 s. The white scale bar is 50 µm.

The intermittent but rhythmic beating of the heart was observed by stage 17 during embryogenesis. The heart proper, a wider posterior portion of dorsal vessel, is ∼55 µm in length with ∼20 µm in diameter (Figure 1). As the heart pumps, the inner diameter of the heart proper oscillates between 5 µm (contraction phase) – 20 µm (relaxation phase) (Figure 2). Anterior to the heart proper is aorta which is a cylindrical tube of ∼200 µm in length with an inner diameter of ∼5 µm (Figure 1).

The live imaging of the embryonic heart clearly shows rhythmic contraction of the cardiac cells in the systolic phase with a time period of ∼150 ms (Figure 2s1–s4 & Figure S1), and the relaxation in the diastolic phase with a time period of ∼700 ms (Figure 2s4-d13-s1 & Figure S1). The period of the single heart beat is, therefore, 850 ms±25 ms, and the ratio of the systolic to diastolic time period is ∼1∶5. A striking feature of the pumping is the precise timing of the closing and opening of the aortic valve Cv1, with respect to the heart proper contraction and dilation (Figure 2). When the cardiac cells contract (s1–s4), the aortic valve Cv1 opens; when the heart cardiac cells relax (d1–d4), check valve Cv1 closes (Figure 2). This dynamic process is also shown in the Movie S1.

An experiment using another GFP line, DMef2::Gal4;Twist::Gal4;UAS::actinGFP, was carried out to further study the dynamics of the behavior of the ostia cells during the pumping (Figure 1 & Movie S2). Movie S2 shows that: 1) three sets of ostia cells open and close simultaneously; 2) They close as the heart proper contracts; and open as the heart proper relaxes. These three sets of ostia cells are presumed to function as another check valve (ostia valves Cv2).

**Figure 3:**
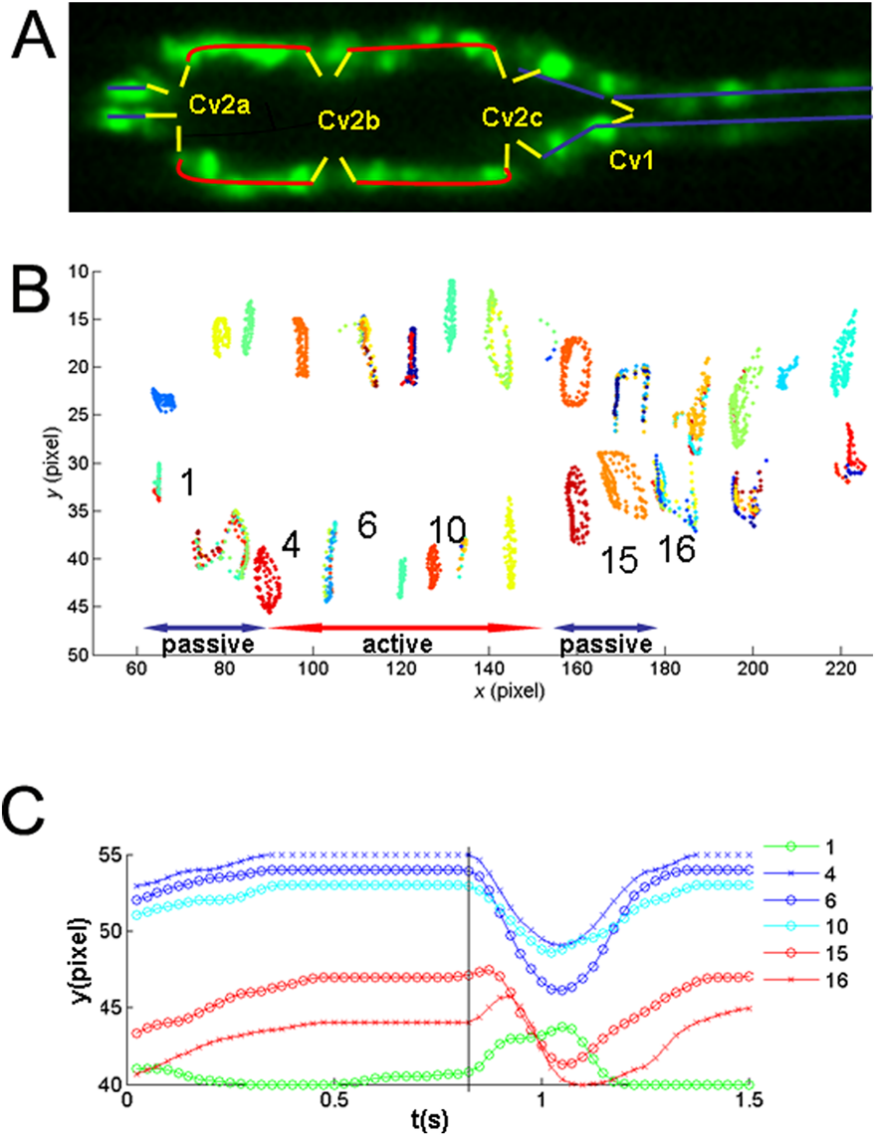
Dynamics of cardiac cell positions of the embryonic *Drosophila* heart. (A) The *Drosophila* embryonic heart tube indicating the active pump (the two rows of cardiac cells, labeled by the red lines), check valves, Cv2a, Cv2b, Cv2c (the three sets of ostia cells) and an aortic valves Cv1. (B) Trajectories of all the tracked cardiac cells during a time period of 5 seconds. The passive regime is identified when the ratio of lateral (x-axis) and radial displacement (y-axis) exceeds 0.3. Each pixel is 0.625 µm. (C) Time evolution of the y position of cardiac cell 1, 4, 6, 10, 15 and 16. Cell numbers are labeled in (B).

To further determine whether such three mechanical components exist in the tubular *Drosophila* heart, we tracked the movement of each cardiac cell using a particle tracking program (Figure 3). The program locates the positions of each cardiac cell by finding the local light intensity maxima, and the positions of each cell are then connected to a track using the nearest-neighbor method. The cells in the middle region of the heart contract actively (active zone), thus only display radial motion along the y-axis; while the cells at the tip and tail of the heart proper move passively (passive zone), this shows up in the lateral motion of the cells along the x-axis (Figure 3B). The analysis of the time evolutions of the radial oscillations of the cardiac cells along various lateral positions shows that the cells in the active zone (4,6,10) contract in synchronization, while the cells in the passive zone are out of phase with the cells in the active zone (Figure 3C). These analyses support the idea that the cardiac cells in an active zone contract in synchronization.

### Identification of a genetic mutation that leads to an aberrant pumping mechanics

**Figure 4:**
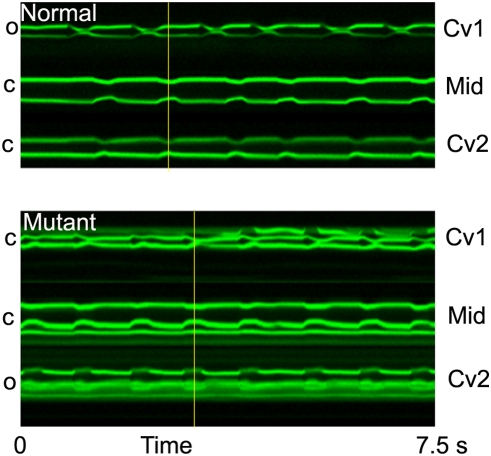
The heart of a VEGFR mutant allele, *Vegf^c2195^*, exhibits reversed sequence of the pumping. Time evolution of the constrictions at the heart tip (representative of Cv2), middle (Heart) and border where heart and aorta meets (Cv1) as shown in Figure 3(A). When the normal heart proper contracts (along the yellow line), Cv1 opens and Cv2 closes to allow fluid to flow from the heart to the aorta. This is not the case for mutant heart. When the mutant heart contracts, Cv1 closes and Cv2 opens, and the fluid is suspected to flow backward from the heart to the open space around the heart.

*Drosophila* has been used extensively as a genetic model organism to uncover molecular basis of development, function and behavior [19], [20]. We have analyzed the dynamics of the pumping of the tubular embryonic heart of *Drosophila*. To gain insight into the molecular mechanism that underlies the pumping mechanics, we searched for mutant *Drosophila* lines that exhibit aberrant pumping mechanics. In examining available mutant *Drosophila* lines for their embryonic heart mechanics, we found a VEGF mutant allele, *Vegf^c2195^*, that exhibits an aberrant pumping mechanics (Figure 4 & Movie S3). The *Vegf^c2195^* embryonic heart forms normal structure and pumps but with the squeezing motion relatively weaker than that of the normal heart, which is accompanied by the apparent reversed sequence of opening and closing of two passive valves, Cv1 and Cv2. In the *Vegf^c2195^* heart, the opening of aortic valve, Cv1, precedes the expansion of the bulb (i.e. the active zone). The expansion of the bulb is followed by the opening of the ostia valves, Cv2. This obvious reversed sequence of the opening/closing of valves and squeezing motion of the Vegf^c2195^ heart also remains in larval stage (Movie S4). This finding suggests the possibility that the VEGF signaling is involved either directly or indirectly in the regulation of pumping mechanics of the *Drosophila* heart.

## Discussion

Herein, we show the dynamics of the opening and closing of the valves in the *Drosophila* embryonic heart (Figure 3). This analysis provides an important information regarding how normal pumping activity of the simple cardiac pump operates. It was previously shown that another relatively simple heart, zebrafish embryonic heart, operates as a dynamic suction pump [6]. Based on our analysis, the *Drosophila* embryonic heart seems to operate differently (Figures 1&2). The zebrafish embryonic heart has bolus chambers and does not have valves; while the *Drosophila* embryonic heart proper is a cylindrical tube with morphologically distinguishable valves along the tube (Figure 1). In the case of *Drosophila* embryonic heart, pumping is realized by the active contraction of the heart proper, and orchestrated opening and closing of the two check valves (Figure 3). In the case of zebrafish embryonic heart, the pumping is driven by the mechanical waves initiated by the contraction of the cardiac cells, and reflected at the locations where the heart bolus meets the inflow or outflow tract [6]. We, further, show that the pumping mechanics of the *Drosophila* embryonic heart is more similar to that of human heart than that of zebrafish (Figure 3). It is also interesting to note that the computed heart ejection rate, using the heart proper volume of two extremes determined by the tracking of each cardiac positions (Figure 3), to be 77.3% (See Methods section for the detailed calculation method that was used). This is slightly higher than the adult human heart ejection rate of 59±7% [21]. It is, however, important to note here that this calculation is based on a situation where we assume that 100% of the hemolymph fluid is ejected and there are no regurgitation, the possibilities that cannot be excluded as we have not been able to directly measure the dynamics of the fluid.

It is not clear why the invertebrate heart functions more similar to that of human than the vertebrate heart does, at least at the level of pumping mechanics. It is possible that this difference may be related to the difference in the developmental time-scale. During zebrafish development, the embryonic heart remains valve-less, but as it matures during postnatal stages, it eventually forms valves and functions similar to the human heart [5]. In contrast to this gradual progression of developmental time-scale in zebrafish, the *Drosophila* heart develops more quickly in that the heart already forms sets of valves during embryogenesis and operates similar to the human heart.

Another question is the role of pumping in *Drosophila* embryo. As the size of *Drosophila* embryo is so small (∼500 µm), the diffusion should be sufficient for the body fluid to be distributed. Also, this small size allows for the sufficient oxygenation of the whole embryo without any active transport systems. In larval and later stages, the extensive network of tracheal system suffices the uniform distribution of oxygen [22]–[24]. It has been proposed that the pumping of the heart in *Drosophila* allows the distribution of hemolymph throughout the body [25]. Although this may be true in larvae and later stages when the body size is too large to rely on simple diffusion of the fluid, the small embryonic size should not require such pumping system of the fluid. We studied whether or not the hemocytes in the hemolymph actually flows through the tubular embryonic heart using the live imaging system, but failed to observe any hemocytes flowing through the embryonic heart during embryonic stage (T.N.S, unpublished result). In fact, it is reported that the movement of hemocytes in embryos are predominantly driven by their active migration [26], [27]. It has been previously shown that the contraction of zebrafish embryonic heart is required for shaping the cardiac structure during development [7]. It is, thus, possible that the pumping of the *Drosophila* embryonic heart is required for the morphogenesis of the developing heart. The third possibility is that the pumping in the embryonic stage allows for the fine-tuning in preparation for the synchronized mechanics of the beating heart in later stages.

If, in fact, the hemolymph is flowing through the embryonic heart of *Drosophila*, how the sequential opening/closing of the valves related to the putative flow mechanics? It has been suggested that the ostia cells (Cv2) form channels for body fluid (hemolymph) to enter into the heart proper [10]. It has been further proposed that Cv1 valve-like structure located between the aorta and the heart proper in coordination with the opening and closing of ostia cells controls unidirectional flow of hemolymph through the dorsal vessel [8], [10]. Our data (Figures 2 & 3, Movies S1&S2) seem to support this proposal. When the cardiac cells in the active zone contract, the aortic valve Cv1 opens to allow fluid to flow from the heart to aorta; when the cardiac cells relax, check valve Cv1 closes to allow for the refill of the heart proper (Figure 2). The validation of this model, however, remains an open question until the dynamics of the hemolymph be directly measured in vivo in the future.

One of the advantage of *Drosophila* as a model system is the power of genetics that is available in this organism. Therefore, we have begun to isolate genetic mutations that affect the mechanics of the cardiac pumping. In this study, we show that the heart of one VEGFR mutant allele, *Vegf^c2195^*, exhibits reverse-sequence of the pumping mechanics (Figure 4). Previously, it has been shown that VEGF signaling pathway is critically involved in cell migration and survival, in particular those of hemocytes in embryos [27]–[32]. It is known that cardiac cells and hemocytes emerge together at the dorsal midline of the developing embryos [33], and it has been reported that the VEGFR is required for hemocytes migration and survival [26]–[28]. Therefore, it is possible that the hemocytes may provide paracrine signals that are required for the function of cardiac cells. However, our preliminary effort to rescue this cardiac phenotype by expressing VEGFR/PVR in hemocytes in developing embryos was not successful (T.N.S., unpublished result). Furthermore, the overexpression of dominant negative form of VEGFR in hemocytes did not result in the cardiac phenotype that we found (T.N.S., unpublished result).

It has been also reported that the mutation in VEGFR results in the defective development of the central nervous system of *Drosophila* embryos [34]. Therefore, it is possible that the aberrant cardiac pumping mechanics found in the *Vegf^c2195^* embryo could be due to the defective neural inputs. Although it is not clear whether the embryonic heart is innervated, it has been previously shown that, at least in adult, the *Drosophila* heart is innervated and the cardiac function is controlled by putative neural inputs [35]. Although this is an intriguing possibility, the neural defect in the VEGFR mutant is presumed to be the consequence of the defective hemocytes migration [34]. Thus, it is difficult to reconcile this possibility with the lack of cardiac phenotype in the embryo where the dominant-negative VEGFR/PVR is expressed in hemocytes. It seems that the VEGFR function required for the cardiac function is not cell autonomous. Although VEGFR/PVR is expressed in cells other than hemocytes, no expression in embryonic cardiac cells is found [27]–[32], [36].

The general idea on how *Drosophila* embryonic heart operates was already documented over a half century ago [25]. However, its mechanics remained ill-defined until now. Our study shown here provides the first set of data that describes the mechanics of the beating function of the embryonic heart in *Drosophila*. Furthermore, we found a genetic mutation that affects the mechanics of the pumping function of the *Drosophila* heart. Availability of a variety of genetic tools in this organism is expected to facilitate further investigations to uncover genetic and molecular basis for the cardiac function, which is also anticipated to have important impact on our further understanding the mechanisms underlying human heart function. We also anticipate that further mechanistic studies of the dynamics of the cardiac pumping and the fluid dynamics of hemolymph contributes to the advancement of our understanding of the design of biological structure and function, and may also potentially provide important information for engineering the cardiac devices in the future.

## Methods

### Fly lines

Toll-GFP, Vegfr^c2195^, Dmef2::Gal4;Twist::Gal4 were kindly provided by Robert Schulz (MD Anderson Cancer Research Institute), Mark Krasnow (Stanford University) and Mary Baylies (Sloan Kettering Research Institute), respectively. UAS-actinGFP line was obtained from Bloomington Drosophila Stock Center.

### Embryo preparation and live imaging

Embryos and larvae were harvested by manually removing the chorion membrane, and they were placed on 35 mm glass bottom culture dish (MatTek Corporation) for imaging. A thin layer of high vacuum grease (Dow Corning) was applied on the surface of glass bottom by a painting brush prior to placing embryos and larvae in order to minimize their movement during the imaging. Once they are placed with their dorsal side down on the culture dish, they were covered by halocarbon oil (95∶5 mix of halocarbon oil 700 and 27, Sigma). The embryos and larvae were imaged by LSM LIVE (Carl Zeiss) in a temperature controlled stage set at 25°C throughout the imaging period. Typically, 5–10 embryos/larvae were placed on a single glass bottom dish for imaging. In each imaging experiment, the images of time series from several embryos and larvae were collected from the single culture dish simultaneously by using the Multi-Time mode system (Carl Zeiss) to minimize any variability from one experiment to another. The comparisons were made from embryos in the same culture dish that were imaged in a single experiment. The pin-hole size was set at 55 µm which allowed us to collect the entire depth of images of beating hearts in live action without collecting z-section series. In all experiments, 5–10 embryos were analyzed and they produced the consistently reproducible cardiac functions.

### Cell tracking and the heart volume calculation

Positions of the cardiac cells are determined using an in house particle tracking software package (download from at http://biofluidics.mae.cornell.edu). Briefly, the positions of the cardiac cells are determined using the locations of the local light intensity maxima. The cross sectional area of the heart at a specific location along the heart tube is evaluated using the distance 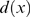 between the positions of the two opposite cardiac cells across the midline of the heart tube, 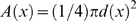. Linear extrapolation is used when the two opposite cells are not exactly at the same x location. The volume of the heart tube is evaluated as 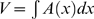. This calculation is repeated for all the images in a movie series, and the results are shown in Figure S1. Ejection rate of the tube heart is (Vmax – Vmin)/Vmax, where Vmax and Vmin are the maximum and minimum volume of the tube heart in one pumping period. We calculated the ejection rate for each of the 11 pumping periods shown in Figure S1. The error of the ejection rate is the standard deviation of these 11 measurements. These calculations are based on the assumption that 100% of the hemolymph fluid is ejected and there are no regurgitation, the possibilities that cannot be ruled out as we have not been able to directly measure the dynamics of the fluid.

## Supporting Information

Figure S1 Time evolution of the tube heart volume as a function of time. The tube heart volume (Y-axis in µm3) was calculated as described in the method section and plotted over time (X-axis in second). An example of systolic (s1-4) and diastolic (d1-13) phases that correspond to the images shown in Figure 2 are indicated at the top.
(0.43 MB TIF)

Movie S1 A 5.00 sec. long movie of the beating heart of a normal transgenic *Drosophila* embryo (Toll-nGFP). Time between two consecutive images is 25 ms and the movie is played in real time. The images are enhanced and colored coded. The red and blue correspond to high and low light intensity respectively. The white circles indicate the locations of the tracked cells.
(20.33 MB AVI)

Movie S2 A movie of normal beating heart of an DMef2::Gal4;Twist::Gal4;UAS::actinGFP *Drosophila* embryo highlighting the opening and closing of the ostia valves (see locations of 6 white arrows) and the aortic valve Cv1. The movie is 8.25 s long, and the time between the consecutive images is 33 ms.
(15.61 MB AVI)

Movie S3 A movie of the heart of a VEGFR mutant allele, *Vegf^c2195^* (embryonic heart). The movie is 6.4 s long and the time between the two consecutive images is 25 ms.
(15.95 MB AVI)

Movie S4 A movie of the heart of a VEGFR mutant allele, *Vegf^c2195^* (larval heart). The movie is 7.8 s long, and the time between the two consecutive images is 25 ms.
(21.95 MB AVI)
