# Supplementary figures and images for: On the Mechanics of Cardiac Function of Drosophila Embryo
Source: PLoS One. 2008 Dec 24;3(12):e4045. doi: 10.1371/journal.pone.0004045 (PMC2602980; doi:10.1371/journal.pone.0004045)

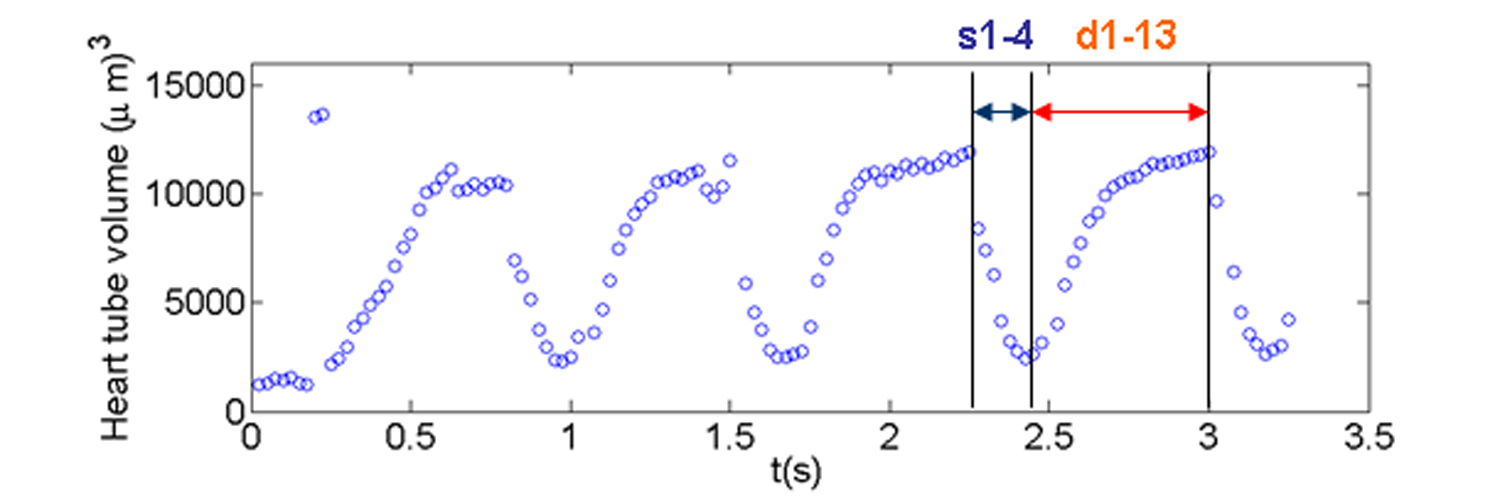

Supplement: Figure S1 — Time evolution of the tube heart volume as a function of time. The tube heart volume (Y-axis in µm3) was calculated as described in the method section and plotted over time (X-axis in second). An example of systolic (s1-4) and diastolic (d1-13) phases that correspond to the images shown in Figure 2 are indicated at the top. (0.43 MB TIF) [file pone.0004045.s001.tif]
